# Surgical Site Infection After Breast Surgery—A Bicentric Retrospective Case–Control Study in Saudi Arabia

**DOI:** 10.3390/clinpract15120231

**Published:** 2025-12-08

**Authors:** Moteb AlSaadi, Salem Alghamdi, Fayyaz Mazari, Sabah Alshuhri, Rustom Bashtawi, Raghad Aljehani, Basmah Alwuqaisi, Rawan Almohammadi, Mahmoud Alfirikh, Sameer Desai, Ebrahim Mahmoud

**Affiliations:** King Faisal Specialist Hospital & Research Centre, Riyadh 12713, Saudi Arabia

**Keywords:** wound infection, surgical site infection, SSI

## Abstract

**Background:** Surgical site infections (SSIs) are common postoperative complications. Data on SSIs following breast surgery in Saudi Arabia are limited because these procedures are not included in the national SSI surveillance system. This study determined the SSI incidence rate, identified associated risk factors, and described the microbiological profiles of patients undergoing breast surgery at two tertiary hospitals in Saudi Arabia. **Methods:** This bicentric retrospective case–control study analyzed 1841 breast surgeries performed at two tertiary hospitals between July 2021 and July 2024. Demographic, surgical, and microbiological data were extracted from electronic medical records. SSIs were defined according to National Healthcare Safety Network criteria. Descriptive statistics summarized patient and surgical characteristics and SSI rates. A matched case–control analysis (1:4 ratio based on age and hospital site) included 172 patients. Multivariable logistic regression was used to identify predictors of SSI. **Results:** The cumulative SSI incidence was 2.4%, and most infections occurred within 30 days of surgery (69%). Gram-negative organisms were predominant in microbiologically positive cases (53.6%), mainly *Klebsiella pneumoniae* and *Pseudomonas aeruginosa*, whereas *Staphylococcus aureus* (including MRSA) accounted for 25%. Immunocompromised status (OR 3.32, 95% CI 1.35–8.14) and surgical drain use (OR 4.07, 95% CI 1.68–9.87) were independently associated with SSI. **Conclusions:** The incidence of SSIs after breast surgery in Saudi Arabia was relatively low. The predominance of Gram-negative pathogens and the identification of immunocompromised status and surgical drain use as major risk factors highlight opportunities for targeted infection prevention strategies. Further studies should validate these findings in larger and more diverse populations and healthcare settings.

## 1. Background

Breast surgeries are associated with the risk of surgical site infection (SSIs), a severe postoperative complication that accounts for more than 30% of all healthcare-associated infections. SSIs are a major cause of healthcare-associated infection worldwide, affecting approximately 2–5% of surgical patients in high-income countries and up to one-third of patients in low- and middle-income countries [1]. These infections can lead to mortality, morbidity, extended hospital stays, frequent emergency visits after discharge, and prolonged recovery periods, which collectively increase treatment costs [2,3,4,5,6]. Preventing SSIs after breast surgery is essential to maintain patient safety and reduce the overall healthcare burden.

SSIs associated with breast surgeries continue to present challenges despite advancements in preventive measures. Reported infection rates vary across hospitals and depend on factors such as patient characteristics, procedure type, and surgical practices. Although breast surgeries are classified as clean (Class 1 wound) procedures with an expectedly low risk of infection, the highest SSI rates have been observed in mastectomy procedures [7,8]. Studies have indicated that the incidence of SSIs in breast surgery ranges from approximately 1% to more than 15% depending on the specific surgical technique [9,10,11,12].

Factors contributing to surgical infection include the complexity of procedures that involve implants and reconstruction [13]. Patient-related characteristics such as obesity, diabetes, smoking, and immunocompromised status can also increase the risk of infection. Gram-positive bacteria, mainly *Staphylococcus aureus*, are the most frequently isolated pathogens from SSIs after breast surgeries. However, Gram-negative bacteria have been reported as the most common isolated pathogen in some studies [14,15,16].

In Saudi Arabia, breast surgeries are not included in the national SSI surveillance system. Therefore, the SSI incidence rate and the contribution of patient- and procedure-related variables in this context remain insufficiently characterized. A review of the local literature identified a lack of focused research in Saudi Arabia examining surgical site infections specifically after breast surgery, highlighting a gap in the current literature. This lack of data limits the development of evidence-based infection prevention protocols and quality improvement initiatives in breast surgery. Addressing this gap through focused epidemiological research can help clarify the SSI burden in the Saudi context, inform tailored infection-control policies, and reduce postoperative complications.

The present study determined the SSI rate among patients undergoing breast surgeries at King Faisal Specialist Hospital and Research Centre (KFSH&RC) in Riyadh and Madinah, Saudi Arabia, and identified risk factors for SSIs. This study also examined the most commonly isolated pathogens from documented SSIs.

## 2. Methods

### 2.1. Study Design and Setting

This retrospective, bicentric study was conducted at KFSH&RC in Riyadh and Madinah, Saudi Arabia, between July 2021 and 2024. KFSH&RC is a major tertiary hospital and research institution that provides advanced medical care and specializes in oncology and complex cases. The Riyadh facility has a capacity of 1100–1500 beds, whereas the Madinah branch has a capacity of 300–400 beds. A matched analysis was performed to evaluate factors associated with SSIs after breast surgery. Each SSI case was matched to four controls (patients without SSIs) based on hospital site and age by using propensity score methods [17]. This study adhered to the Strengthening the Reporting of Observational Studies in Epidemiology (STROBE) guidelines for case–control studies.

### 2.2. Inclusion Criteria

All adult patients (aged ≥18 years) who underwent breast surgery at KFSH&RC Riyadh and Madinah were included.

### 2.3. Exclusion Criteria

Patients who had an active infection at the intended surgical area at the time of surgery were excluded.

### 2.4. Sample Size and Propensity Score Matching

A total of 1841 breast surgeries were performed during the 3-year study period. Propensity score methods were used to evaluate the risk factors associated with SSIs after breast surgery. A logistic regression model was used to estimate each patient’s probability of developing an SSI, with hospital site and age included as predictor variables. The resulting propensity scores were then used to conduct 1:4 nearest-neighbor matching without replacement by using calipers set at 0.2 standard deviations of the estimated propensity score [17]. After the application of exclusion criteria and completion of the matching process, the final sample included 172 patients, comprising 36 cases and 136 controls.

### 2.5. Data Collection

Data were collected from the hospital’s electronic medical records. The variables of interest included patient-related factors such as obesity, diabetes, age, smoking, body mass index (BMI), and immunocompromised status, as well as surgical factors such as the type of surgery, American Society of Anesthesiologists (ASA) score, wound class, surgery duration, and use of drains and antibiotic prophylaxis. These factors were selected on the basis of the literature indicating their potential impact on SSI rates [14,16,18,19,20].

Immunocompromised status was defined in accordance with CDC/National Healthcare Safety Network (NHSN) criteria as the presence of one or more conditions associated with impaired host defense. These conditions included primary or acquired immunodeficiency, a history of solid organ or bone marrow transplantation, hematologic malignancy, active chemotherapy or radiotherapy, a history of splenectomy, or corticosteroid therapy exceeding 20 mg/day for at least 2 weeks. Patients meeting any of these criteria were classified as immunocompromised for the analysis [7].

### 2.6. Microbiological Assessment

The microbiology of the SSIs was assessed using wound cultures obtained during the clinical diagnosis of infection. All samples were examined in the hospital’s microbiology laboratory, where organisms were identified through Gram staining and standard culture methods.

### 2.7. SSI Definition

SSI was defined in accordance with the NHSN guidelines, which classify surgical infections as superficial incisional, deep incisional, or organ/space infections based on clinical and microbiological findings [7]. Data for this study were obtained from the hospital’s infection control records, which are maintained in accordance with NHSN criteria. These records include information collected through routine postoperative surveillance.

### 2.8. Perioperative Antibiotic Prophylaxis

All eligible patients were managed in accordance with the hospital’s perioperative antibiotic prophylaxis protocol in place during the study period. The regimen consisted of administering a first-generation cephalosporin, typically cefazolin 2 g intravenously, within 60 min before surgical incision. Clindamycin was used as the alternative agent for patients with a documented β-lactam allergy. Intraoperative redosing was performed for procedures lasting longer than 4 h or in cases of significant blood loss exceeding 1.5 L. This protocol reflects the hospital’s routine surgical prophylaxis practices during the period in which the procedures were performed.

### 2.9. Statistical Analysis

Descriptive statistics were used to summarize patient demographics, surgical characteristics, and SSI rates. The SSI rate was calculated by dividing the number of documented SSIs by the total number of surgeries performed during the study period.

Factors associated with the occurrence of SSI were evaluated using univariable logistic regression analyses. Variables with a *p* value of <0.20 in the univariable analyses were included in the multivariable model. A backward stepwise selection approach based on the Akaike information criterion (AIC) was used to identify the most predictive variables. This approach involves sequentially removing variables that contribute least to the model, thereby optimizing the balance between model complexity and explanatory value. Model results are reported as odds ratios (ORs) with 95% confidence intervals, and statistical significance was set at 0.05. Missing data were minimal (<5% for diabetes status). All statistical analyses were performed using R version 4.1.1 [21].

## 3. Results

### 3.1. Study Population

A total of 1841 breast procedures were performed during the study period, and 86% of these procedures were conducted at KFSH&RC Riyadh. The most common procedures were mastectomy (57%) and the removal of a breast mass or lump (33%). Aesthetic or oncoplastic surgeries accounted for 4.4% of procedures, and breast implant surgeries accounted for 5.8%. Approximately 0.5% of patients underwent surgery for gynecomastia.

### 3.2. Incidence of SSI

The overall cumulative incidence of SSI was 2.4% (44/1841). Breast implant procedures (9.4%) and aesthetic or oncoplastic surgeries (4.9%) had the highest SSI rates.

**Figure 1:**
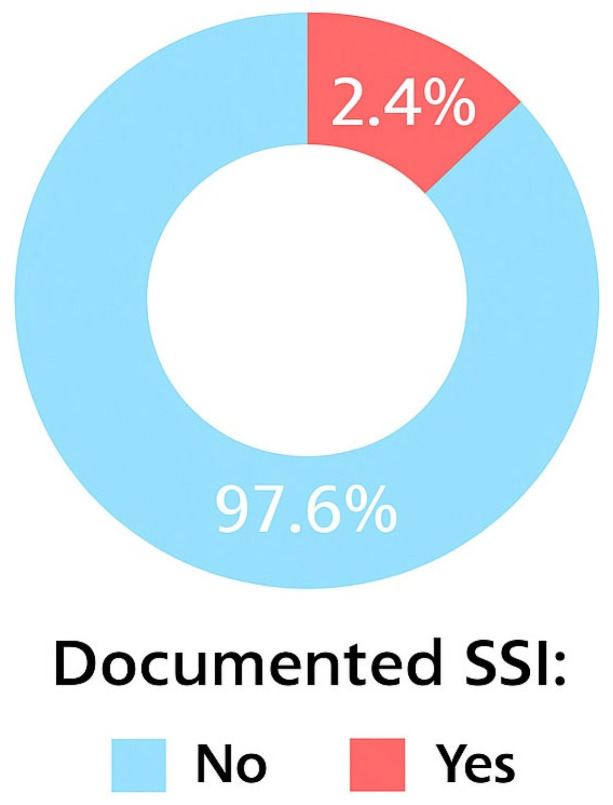
Cumulative incidence of SSIs.

**Figure 2:**
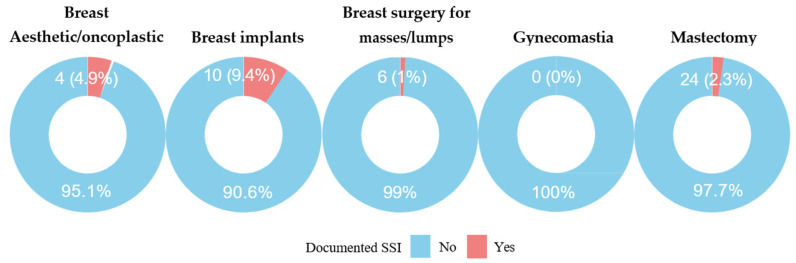
SSI per procedure type.

Breast implant procedures had the greatest SSI risk, with approximately 10% of surgeries involving implants resulting in postoperative infection. Aesthetic breast surgeries had an infection rate of approximately 5%. In comparison, mastectomy and breast mass removal procedures had substantially lower infection rates of 1.0% and 2.3%, respectively (Figure 1 and Figure 2).

### 3.3. Matched Propensity Score Analysis of Factors Associated with SSIs

A matched analysis was conducted to identify risk factors associated with SSIs. The final matched sample included 172 patients (36 cases and 136 controls), matched in a 1:4 ratio by hospital site and age. Table 1 summarizes the baseline characteristics of the matched cohort and presents the corresponding standardized mean differences (SMDs) used to assess covariate balance. The median age was similar between cases (53 years, IQR 45–63 years) and controls (55 years, IQR 45–63 years), with an SMD of −0.04, indicating excellent balance. Body mass index was modestly higher among cases (median 32.3 kg/m^2^, IQR 24.8–36.0 kg/m^2^) compared with controls (29.9 kg/m^2^, IQR 26.7–34.6 kg/m^2^), indicated by an SMD of 0.19. The distribution by hospital site was nearly identical, with 83% of cases and 84% of controls treated at the Riyadh facility (SMD = 0.01). Diabetes was more common in cases (33.3%) than in controls (24.4%), although the imbalance was minimal (SMD = 0.09).

ASA physical status was similarly distributed between groups (SMD = 0.04), and most patients were classified as ASA II–III. None of the cases were classified as ASA I. Wound classification according to NHSN criteria was largely comparable, although clean-contaminated wounds were more frequent among cases (61%) than controls (57%; SMD = 0.25). Nearly all patients received antibiotic prophylaxis (97.2% of cases and 100% of controls) as per hospital protocol. Surgical drain use exhibited substantial imbalance, with drains used in 77.8% of cases compared with 41.2% of controls (SMD = 0.37). The median duration of drain placement was also considerably longer among cases (16.5 days, IQR 8.3–23.3 days) than among controls (8.0 days, IQR 6.0–14.5 days).

Surgery duration was also longer among cases (median 126 min, IQR 96–178 min) than among controls (97 min, IQR 70–148 min), with an SMD of 0.50, indicating moderate imbalance. Among the 80 patients classified as immunocompromised, 64 (80%) were on active chemotherapy or radiotherapy before surgery. This subgroup formed the largest proportion of patients in the immunocompromised category.

Table 2 presents the results of the univariable analyses. The variables that met the inclusion threshold for the multivariable model (*p* < 0.20) were MRSA infection or colonization before surgery (OR: 9.50; 95% CI: 1.58–77.8; *p* = 0.018), use of surgical drains (OR: 5.00; 95% CI: 2.21–12.5; *p* < 0.001), immunocompromised status (OR: 3.95; 95% CI: 1.81–9.21; *p* < 0.001), malignant tumors (OR: 2.14; 95% CI: 1.00–4.86; *p* = 0.057), and longer surgery duration (OR: 1.01; 95% CI: 1.00–1.01; *p* = 0.007). In the multivariable model, immunocompromised status and surgical drain use were independently associated with SSI. Immunocompromised patients had 3.32 times higher odds (95% CI: 1.35–8.14) of developing an SSI compared with non-immunocompromised patients. Patients with surgical drains had 4.07 times the odds (95% CI: 1.68–9.87) of developing an SSI compared with those without drains (Table 3).

### 3.4. Microbiology of SSIs

Among the matched cases with positive microbiological results (*n* = 28), *Staphylococcus aureus* and *Klebsiella pneumoniae* were the most frequently identified organisms, each identified in 5 cases (14%). *Pseudomonas aeruginosa* and MRSA were each isolated in four cases (11%). Mixed Gram-positive and Gram-negative organisms were detected in two cases (5.6%). Overall, Gram-negative organisms accounted for about 53.6% of all positive microbiological results, whereas Staphylococcus aureus (including MRSA) accounted for 25% (Table 4).

### 3.5. Postoperative Onset of SSIs

SSI cases were categorized on the basis of the time of onset as early-onset or late-onset infections. Infections diagnosed within 30 days of the surgical procedure were classified as early-onset, whereas infections identified more than 30 days after surgery were classified as late-onset. Among the 36 documented SSIs, 25 cases (69.4%) were early-onset and 11 cases (30.6%) were late-onset.

### 3.6. Duration of Surgical Drain in Place

The mean duration of surgical drain placement was 11.7 days, and the median was 10 days (IQR 6–16). Drain duration was longer among cases (median 16.5 days, IQR 8.3–23.3) than among controls (8.0 days, IQR 6.0–14.5). Most patients with available drain-duration data (*n* = 49, 69%) had drains removed within one to two weeks after surgery, which aligns with common postoperative practice. A smaller proportion required less than one week of drainage (*n* = 14, 20%). Drain duration exceeding three weeks was observed in eight patients (11%).

### 3.7. Outcome of SSIs

Among the 36 documented SSIs, 22 (61.1%) cases had no long-term clinical consequences. Reoperation was required in 12 patients (33.3%) because of infection-related complications. In two cases (5.6%), the SSI affected the patient’s oncological treatment plan, necessitating a change in treatment timing.

## 4. Discussion

This study explored the epidemiology and risk factors of SSI following breast surgery at two tertiary hospitals in Saudi Arabia. The overall cumulative incidence rate of SSI in this cohort was 2.4%, with variation observed across the different types of breast surgery.

Although local data are limited, the SSI rate identified in this cohort is lower than rates reported in other countries. For example, a cohort study conducted in Poland that included 2129 surgical interventions reported a higher incidence of 6.2% [19].

Breast implants and aesthetic or oncoplastic surgeries had the highest SSI rates in this study (9.4% and 4.9%, respectively), whereas mastectomy and breast mass removal procedures had lower rates. These findings are consistent with previous studies showing that breast reconstruction, especially implant-based procedures, has a high risk of SSIs. Olsen et al. reported an SSI rate of 5.0% following mastectomy alone, which increased to 10.3% when the procedure included implant-based reconstruction [20]. Additional evidence suggests that chronic inflammation associated with late breast implant seromas may be linked to bacterial contamination, particularly in cases involving textured implants [22]. The increased surface area of textured implants may promote bacterial adhesion and biofilm formation, contributing to persistent inflammation and seroma development. These observations highlight the importance of implementing infection prevention strategies tailored to the type of breast surgery performed.

In this study, *S. aureus* and MRSA together accounted for 25% of identified microorganisms, reflecting the ongoing clinical relevance of *S. aureus*, particularly its resistant strains. Gram-negative organisms represented 53.6% of positive microbiological results and primarily included *K. pneumoniae*, *P. aeruginosa*, and *Enterobacter cloacae*. Although many studies describe a predominance of Gram-positive isolates in breast surgery–related SSIs, the present findings align with reports indicating that Gram-negative organisms may be more common in some settings. For example, Mukhtar et al. identified Gram-negative bacteria as the most frequently isolated pathogens [23]. The high isolation of Gram-negative bacteria might reflect a nosocomial origin, which highlights the need for enhancing infection control measures. This may also be influenced by the presence of breast implants, which can serve as surfaces for biofilm formation. Previous studies have suggested an elevated likelihood of implant infections caused by Gram-negative organisms [24,25].

This study identified the immunocompromised status of patients and the use of surgical drains as the major predictors of SSI after breast surgery. This finding is consistent with evidence indicating that immunocompromised status, whether related to corticosteroid therapy, chemotherapy, radiotherapy, or underlying medical conditions, markedly increases the risk of postoperative infection [26]. In this cohort, chemotherapy or radiotherapy exposure was the main contributor to immunocompromised status, accounting for 80% of cases. This finding highlights the particular vulnerability of oncology patients undergoing breast surgery and supports prior studies that systemic cancer therapy substantially increases postoperative infection risk [27,28,29]. These findings demonstrate the importance of applying tailored infection prevention and control strategies for immunocompromised populations.

Surgical drain use, which is primarily intended to prevent seroma formation, was also a significant risk factor for SSI. This aligns with the established knowledge that drains serve as an entry route for microorganisms to the wound, particularly if not well managed. Given the association between some breast surgery techniques and increased seroma formation, extended drain duration (over 15 days on average) may be unavoidable in some cases, further increasing infection risk [30]. In this study, the median duration of surgical drain was 10 days. Previous studies have recommended drain removal either once the output falls below 50 mL over 48 h or at predetermined intervals of less than a week in general [31,32,33,34]. Strategies to safely shorten drain duration warrant further investigation because earlier removal may reduce infection risk.

Obesity and diabetes were not found to be predictors of SSI in this study, although both factors have been implicated in the increased risk of infection in general as well as breast surgeries. The overall low SSI rate in this cohort may have limited the ability to detect associations between these variables and postoperative infection. Smoking was excluded from the analysis because no patients in either group were documented as smokers, which may reflect the low prevalence of smoking among women in Saudi Arabia or possible under-documentation in the medical records [35].

Approximately 70% of SSIs occurred within 30 days of surgery, whereas 30% occurred beyond this period. This pattern is consistent with the breast surgery literature, where most SSIs are identified during the first postoperative month, particularly after mastectomy and reconstruction [19,36]. Late-onset infections were more frequently associated with implants, consistent with NHSN surveillance windows, which extend to 30 days for superficial SSIs, 90 days for deep or organ/space SSIs, and up to one year for procedures involving implants [7].

Findings from this study highlight the importance of implementing preventive protocols tailored to breast surgery, particularly in settings with limited local data. Future research should focus on developing and validating SSI risk prediction models specifically designed for patients undergoing breast surgery.

Although this study provides key insights into SSIs in a specific population and adds to the limited local literature in Saudi Arabia, several limitations should be considered. The retrospective design may introduce bias related to missing or incomplete data. For example, documentation of skin preparation, surgical drain management, antibiotic prophylaxis, and wound care suggested optimal practice in almost all patients, but reliance on chart records may not fully capture variability in clinical practice. Another limitation is the matching approach, which included only age and hospital site. Although this method balanced these two key variables, it may have introduced selection bias and potentially skewed the study findings. Clinically relevant factors such as ASA score and BMI were not retained in the multivariable model because they did not meet criteria for statistical significance, and other covariates, including procedure type, surgery duration, and drain use, showed residual imbalance after matching. These issues may contribute to residual confounding, and the results should therefore be interpreted with caution.

## 5. Conclusions

The incidence of SSIs following breast surgery in Saudi Arabia appears to be lower compared with rates reported in other countries, although available local data remain limited. A notable finding of this study is the predominance of Gram-negative isolates. Factors such as immunocompromised status and surgical drain use represent important considerations for strengthening infection prevention strategies. The generalizability of these findings may be restricted because the study was conducted in specialized tertiary centers with established protocols. Prospective studies in more diverse populations and healthcare settings are warranted to validate these observations.

## Figures and Tables

**Table 1:** Baseline Characteristics of Matched Cases and Controls (*n* = 172).

	Case, *n* = 36 ^1^	Control, *n* = 136 ^1^	Difference ^2^
Patient age at surgery, years	53 (45, 63)	55 (45, 63)	−0.04
BMI, kg/m^2^	32.3 (24.8, 36.0)	29.9 (26.7, 34.6)	0.19
Hospital			0.01
MADINAH	6 (17%)	22 (16%)	
RIYADH	30 (83%)	114 (84%)	
Diabetic	12 (33.3%)	33 (24.4%)	0.09
ASA score			0.04
1	0 (0%)	4 (2.9%)	
2	17 (47%)	59 (43%)	
3	19 (53%)	73 (54%)	
NHSN Index			0.25
Clean	14 (39%)	58 (43%)	
Clean-Contaminated	22 (61%)	78 (57%)	
Type of breast surgery			1.6
Breast aesthetic or oncoplastic surgeries	4 (11%)	3 (2.2%)	
Breast implants	7 (19%)	36 (26%)	
Breast surgery for masses/lumps	4 (11%)	85 (63%)	
Mastectomy	21 (58%)	12 (8.8%)	
Use of surgical drain	28 (77.8%)	56 (41.2%)	0.37
Surgery duration, minutes	126 (96, 178)	97 (70, 148)	0.50

^1^ Median (IQR); *n* (%). ^2^ Standardized Mean Difference.

**Table 2:** Univariable screening for factors associated with SSI.

Characteristic	*n*	OR	95% CI	*p* Value
Body mass index	172	1.03	0.98, 1.08	0.30
ASA score	172	1.07	0.55, 2.15	0.84
Diabetic	171	—	—	—
No		—	—	—
Yes		1.55	0.68, 3.39	0.28
Malignant tumor	172	—	—	—
No		—	—	—
Yes		2.14	1.00, 4.86	0.057
Immunocompromised	172	—	—	—
No		—	—	—
Yes		3.95	1.81, 9.21	<0.001
MRSA infection or colonization before surgery	172	—	—	—
No		—	—	—
Unknown		1.19	0.51, 3.03	0.70
Yes		9.50	1.58, 77.8	0.018
Surgery duration (minutes)	172	1.01	1.00, 1.01	0.007
Use of surgical drain	172	—	—	—
No		—	—	—
Yes		5.00	2.21, 12.5	<0.001

*n* = Number, OR = Odds Ratio, CI = Confidence Interval.

**Table 3:** Multivariable model for factors associated with SSIs.

Factor	OR	95% CI	*p* Value
Immunocompromised			
No	-	-	
Yes	3.32	1.35, 8.14	0.009
Use of surgical drain			
No	-	-	
Yes	4.07	1.68, 9.87	0.002

OR = Odds Ratio, Cl = Confidence Interval.

**Table 4:** Microbiological Results.

Microbiological Results	Number	Percentage
*Corynebacterium* spp.	1	2.8%
Gram-positive cocci	1	2.8%
*Streptococcus agalactiae* (Group B)	1	2.8%
Mixed Gram-positive and mixed Gram-negative organisms	2	5.6%
*Serratia marcescens*	2	5.6%
*Enterobacter cloacae*	3	8.3%
Methicillin-resistant *Staphylococcus aureus* (MRSA)	4	11%
*Pseudomonas aeruginosa*	4	11%
*Klebsiella pneumoniae*	5	14%
*Staphylococcus aureus*	5	14%
No organism	8	22%

## Data Availability

The data presented in this study are available on reasonable request from the corresponding author.
